# Effectiveness of biostimulation through nutrient content on the bioremediation of phenanthrene contaminated soil

**DOI:** 10.1186/s40201-014-0143-1

**Published:** 2014-12-24

**Authors:** Roshanak Rezaei Kalantary, Anoushiravan Mohseni-Bandpi, Ali Esrafili, Simin Nasseri, Fatemeh Rashid Ashmagh, Sahand Jorfi, Mahsa Ja’fari

**Affiliations:** Department of Environmental Health Engineering, School of Public Health, Iran University of Medical Sciences, Tehran, Iran; Department of Environmental Health Engineering, School of Public Health, Shahid Beheshti University of Medical Sciences, Tehran, Iran; Department of Environmental Health Engineering, School of Public Health and Center for Water Quality Research, Institute forEnvironmental Research, Tehran University of Medical Sciences, Tehran, Iran; Environmental Technology Research Center, Ahvaz Jondishapour University of MedicalSciences, Ahvaz, Iran; School of Public Health, Ahvaz Jondishapour University of MedicalSciences, Ahvaz, Iran; Petroleum University of technology, Abadan faculty of petroleum engineering, Abadan, Iran

**Keywords:** Polycyclic aromatic hydrocarbons, Phenanthrene, Bioremediation, Biostimulation, Macro/Micro nutrient

## Abstract

Bioremediation has shown its applicability for removal of polycyclic aromatic hydrocarbons (PAHs) from soil and sediments. In the present study, the effect of biostimulation on phenanthrene removal from contaminated soil via adding macro and/or micronutrients and trace elements was investigated. For these purposes three macro nutrients (as N, P and K), eight micronutrients (as Mg, S, Fe, Cl, Zn, Mn, Cu and Na) and four trace elements (as B, Mo, Co and Ni) in 11 mineral salts (MS) as variables were used. Placket-Burman statistical design was used to evaluate significance of variables (MS) in two levels of high and low. A consortium of adapted microorganisms with PAHs was used for inoculation to the soil slurry which was spiked with phenanthrene in concentration of 500 mg/kg soil. The optimal reduction resulted when a high level of macro nutrient in the range of 67-87% and low level of micro nutrient in the range of 12-32% were used with the nitrogen as the dominant macronutrient. The Pareto chart showed that NH_4_NO_3_ was the most effective variable in this experiment. The effect of elements on phenanthrene biodegradation showed following sequence as N > K > P > Cl > Na > Mg. Effectiveness of the other elements in all runs was less than 1%. The type and concentration of nutrient can play an important role in biodegradation of phenanthrene. Biostimulation with suitable combination of nutrient can enhance bioremediation of PAHs contaminated soils.

## Background

Polycyclic aromatic hydrocarbons (PAHs) are chemical compounds made up of more than two fused aromatic rings in a linear or clustered arrangement, usually containing only carbon (C) and hydrogen (H) atoms, although nitrogen (N), sulphur (S) and oxygen (O) atoms may readily substitute in the benzene ring to form heterocyclic aromatic compounds. They are produced due to incomplete combustion of hydrocarbons and fossil fuels. Furthermore, natural occurrences also contribute in PAHs production. PAHs are highly hydrophobic which make them persistent and toxic to the environment and human [1,2]. Soil contamination to PAHs causes great health concerns because their persistency, toxicity, mutagenicity and carcinogenicity effects have been proved [3]. Different approaches like solvent extraction [4], phytoremediation [5], chemical remediation with various oxidants [6], photocatalytic remediation [7], electrochemical remediation [8], thermal destruction [9] and microbial degradation (bioremediation) [10,11] have been experimented for removal of PAHs from contaminated soils which bioremediation has been considered the most suitable for remediation of soils contaminated to PAHs [3,12]. Being environmental friendly and less ecologically damaging, less physical, chemical and biological changes in environmental conditions, less addition of chemicals, lower operational costs and proven efficiency are the main advantageous of bioremediation [13]. Both physical and chemical factors of reaction medium are effective on process efficiency. These factors include temperature, pH and accessibility of substrate to microorganisms, oxygen, nutrients, presence of electron acceptors and addition of macro and micro elements. Addition of nutrients, macro and micro elements or oxygen to the polluted site to enhance the microbial degradation ability is called biostimulation. According to literature, two processes including biostimulation and bioaugmentation are usually considered to enhance the bioremediation of soils contaminated by hydrocarbons. Biostimulation increases the bacterial activity of various strains present in the contaminated soil through the addition of nutrients [14], humic compounds [15] or other chemicals which could affect on the bacterial condition.

The needs of bacteria and other microorganisms to nutrients are approximately similar to their cell composition. Three main categories of nutrients for microbial metabolism (macro and micro nutrient, and trace elements) were studied to determine the best nutritional composition in bioremediation of PAHs contaminated soils. However, carbon is usually needed in higher amounts and can be provided by target pollutants. Bailey and Ollis (1986) said that nitrogen and phosphorus as macronutrients are 14% and 3% of dry weight of a typical microbial cell, respectively [16] but Liebeg and Cutright (1999) reported in their research that phosphorus was the dominant macronutrient in bioremediation of PAH. However micronutrients such as sulfur, calcium and magnesium in microbial cell are only 1, 0.5, and 0.5%, respectively [17], but the concentration of these micronutrient and the others in mineral salt medium for bioremediation were very different. For investigating the effect of several macro, micro and trace nutrients the Plackett–Burman experimental design was used in optimization of liquid culture medium because of its potential in considering many variables.

Selection of the most efficient nutritional mixture can be investigated by the experimental or statistical approaches. Statistical experimental designs have some advantages which directed researchers to consider those in their bio studies such as their reliability, time saving (being rapid), cost saving and their reduction in the total number of experiments [18]. Different experimental design approaches are developed for such optimization of process conditions. Approaches like multi factorial designs are difficult because high number of variables should be screened. Also the orthogonal nature of Plackett–Burman gives pure effect of each variable [18]. Many studies are implemented according to Plackett–Burman experimental design. Chauhan et al. (2007) used Plackett–Burman statistical design for lactic acid production by *Lactobacillus sp. KCP01* using date juice [18]. Zhou et al. (2011) studied phenol degradation according to Plackett–Burman experimental design [19]. In the current study, the addition of different macro and/or micronutrients and trace elements in mineral salts medium (MSM) for phenanthrene removal from contaminated soil sample was experimented according to Plackett–Burman experimental.

## Materials and methods

### Chemicals

Acetone, methanol and acetonitril in HPLC grade were purchased from ROMIL Company. Phenanthrene (Purity > 98%), the salts for nutrient solutions were purchased from Fluka, Sigma Aldrich and Merck Company. Nutrient Broth and R_2_A Agar were supplied by Difco and BIOMARK Company respectively.

### Phenanthrene biodegradation investigation

Soil was collected from a depth of 5–20 cm of ground’s surface and was passed through a 2-mm sieve. To get free of any organic matter it was washed with acetone several times and then distilled water was used for removing residual acetone. The soil was consisted of 83.1% sand, 11.9% silt and 5% clay. Total nitrogen and phosphorus were 0.025% and 0.0012%, respectively. The pH and electrical conductivity (EC) of the soil were 7.4 and 3.2 ds/m, respectively.

Two grams of dry soil was placed into a 50 mL Erlenmeyer flask as bioreactor. The bioreactors containing clean soil were autoclaved. A measured weight of phenanthrene was dissolved in acetone then it was used to spike the soil to have 500 mg phenanthrene/kg dry soil. For evaporation of the residual acetone the bioreactors were placed in a shaker (Heidolph, ProMax 2020 model) at the velocity of 180 rpm in room temperature and dark condition to have a uniform dispersion of phenanthrene.

The soil was inoculated with a culture of bacteria with an optical density of 1 at 630 nm [20] using CECIL UV/vis spectrophotometer (model 7100) in different concentrations of nutrients according to Table 1. The culture consisted of five types of bacteria; *Bacillus sporogenes, Bacillus licheniformis, Capnocytophaga ochracea* (presumably)*, Acinetobacter sporogenes* and *Staphylococcus xylosus* which enriched with Phenanthrene in our previous study [21]. At the end, the soil liquid ratio was 10% w/v and all the samples and their similar blanks were put in the shaker at the velocity of 180 rpm in the room temperature (22 ± 3°C) with pH adjusted at 6.8 ± 0.2 for 8 weeks.

**Table 1:** **Twelve-trial Plackett–Burman design to study eleven factors in phenanthrene removal from soil: a comparison of experimented and predicted removal** [22]

**Run**	**A**	**B**	**C**	**D**	**E**	**F**	**G**	**H**	**I**	**J**	**K**
	**KH**_**2**_ **PO**_**4**_	**K**_**2**_ **HPO**_**4**_	**NH**_**4**_ **NO**_**3**_	**MgSO**_**4**_	**FeCL**_**3**_	**NaCl**	**ZnSO**_**4**_ **.H**_**2**_ **O**	**MnSO**_**4**_ **.H**_**2**_ **O**	**CuSO**_**4**_ **.5H**_**2**_ **O**	**FeSO**_**4**_ **.7H**_**2**_ **O**	**Trace elements**
1	**+**	**-**	**+**	**-**	**-**	**-**	**+**	**+**	**+**	**-**	**+**
2	**+**	**+**	**-**	**+**	**-**	**-**	**-**	**+**	**+**	**+**	**-**
3	**-**	**+**	**+**	**-**	**+**	**-**	**-**	**-**	**+**	**+**	**+**
4	**+**	**-**	**+**	**+**	**-**	**+**	**-**	**-**	**-**	**+**	**+**
5	**+**	**+**	**-**	**+**	**+**	**-**	**+**	**-**	**-**	**-**	**+**
6	**+**	**+**	**+**	**-**	**+**	**+**	**-**	**+**	**-**	**-**	**-**
7	**-**	**+**	**+**	**+**	**-**	**+**	**+**	**-**	**+**	**-**	**-**
8	**-**	**-**	**+**	**+**	**+**	**-**	**+**	**+**	**-**	**+**	**-**
9	**-**	**-**	**-**	**+**	**+**	**+**	**-**	**+**	**+**	**-**	**+**
10	**+**	**-**	**-**	**-**	**+**	**+**	**+**	**-**	**+**	**+**	**-**
11	**-**	**+**	**-**	**-**	**-**	**+**	**+**	**+**	**-**	**+**	**+**
12	**-**	**-**	**-**	**-**	**-**	**-**	**-**	**-**	**-**	**-**	**-**

### Experimental design

The liquid medium was processed for assessing the biodegradation of phenanthrene. Parametric optimization for biodegradation was studied with respect to three macro nutrients (as N, P and K), eight micronutrients (as Mg, S, Fe, Cl, Zn, Mn, Cu and Na) and four trace elements (as B, Mo, Co and Ni) in mineral salt mediums. For this purpose eleven mineral salts were used according to the literature [23].

Plackett-Burman design is an efficient method to identify the important factors among a large number of variables. In this study, a 12 runs Plackett-Burman design was used to screen the important variables that significantly influenced phenanthrene degradation.

Each variable was applied at two levels of high (+) and low (−). The corresponding amount of variables and the level of them in 12 trials were shown in Tables 1, 2 and 3.

**Table 2:** **Variables showing medium components used in Plackett–Burman design**

**Factor**	**Variables**	**Maximum level g/L**	**Minimum level g/L**
**A**	KH_2_PO_4_	3	0.5
**B**	K_2_HPO_4_	3	0.5
**C**	NH_4_NO_3_	6.1	0.4
**D**	MgSO_4_	0.5	0.1
**E**	FeCL_3_	0.2	0.01
**F**	NaCl	0.8	0.01
**G**	ZnSO_4_.H_2_O	0.00005	0.02
**H**	MnSO_4_.H_2_O	0.004	0.0002
**I**	CuSO_4_.5H_2_O	0.0004	0.00002
**J**	FeSO_4_.7H_2_O	0.001	0.1
**K**	Trace elements	1 mL	1 mL

**Table 3:** **The trace elements of nutrient solutions**

**Salts for trace elements**	**Maximum level (+) g/L**	**Minimum level (−) g/L**
H_3_BO_3_	13 × 10 ^−3^	5 × 10 ^−3^
Na_2_MoO_4_	1 × 10 ^−5^	1.4 × 10 ^−6^
CoCl_2_	1 × 10 ^−4^	1 × 10 ^−4^
NiCl_2_	2 × 10 ^−4^	2 × 10 ^−4^

### Determination of microbial population

The population of the inoculated culture was determined by the most probable number (MPN) method. The bacterial suspension was diluted tenfold serially in a sterile ringer solution (8.5 g NaCl per 1 L DW) and added to the sterile Nutrient Broth in the ratio of 10% of volume in triplicates in five series then they were incubated in 30°C. After 48 hours the turbidity of positive growth was seen in direct observation. The population of bacterial consortium was estimated according to statistical table of MPN [21].

### The bioremediation efficiency in naturally contaminated soil

In order to investigate the optimized process efficiency, a soil sample naturally contaminated to PAHs was transferred to lab and the bioremediation efficiency was tried for, according to the most efficient expremental results and optimized conditions exactly like section 2–2. The preliminary investigations by GC-MS revealed that the soil was contaminated to phenenthrene, pyrene, anthracene, flourene and different aliphatic hydrocarbons. After 8 weeks the removal efficiency of PAHs was determined.

### Extraction and analysis

The residual phenanthrene in the soil was extracted with methanol by ultrasonic homogenizer (Bandelin Sonoplus HD 2070) according to EPA 3550B (EPA) [24]. The extracted sample was then centrifuged (Hettich D7200) for 15 minutes at 6000 rpm , filtered through 2–3 cm of glass wool and then A portion of the filtered solution was used for analysis.

The extract was quantified by a HPLC from CECIL Company equipped with an Adept CE 4100 dual piston high pressure solvent delivery pump, a sample injection valve having a 20 μL sample loop and an Adept CE 4201 UV-Visible variable wavelength detector with 8 μL × 10 mm flow cell set at the wavelength of 220 nm. Separations were carried out on a C18 column, and the mobile phase was a mixture of methanol/deionized (90:10, v/v). The flow rate was 1.0 mL/min and the retention time of phenanthrene was 10.0 min. The concentration of phenanthrene was determined after the calibration of the method with standard phenanthrene samples.

## Results

### The phenanthrene removal efficiency in different nutrient solutions

**Figure 1:**
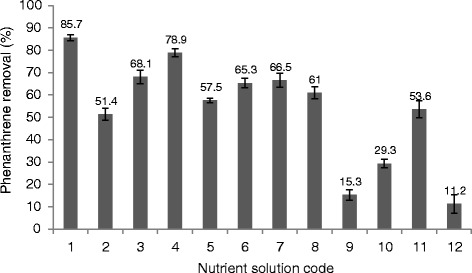
**Removal efficiency of phenanthrene for various nutrient solutions.**

The results of average phenanthrene removal for different nutrient solutions are presented in Figure 1. The most removal efficiency of 85.7% was observed for run 1 with higher amount of nitrogen, phosphorus, Zn, Mn and trace elements in liquid medium folloewd by number 4, 3, 7 and 8 with removal values of 78.9%, 68.1%, 66.5% and 65.3% respectively.

### Individual effect of factors on phenanthrene removal

**Figure 2:**
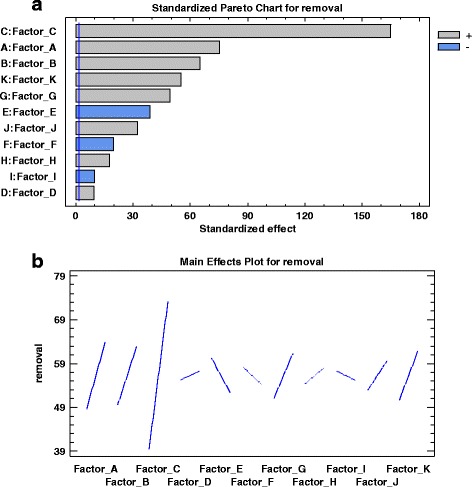
**The individual effect of each factor on phenanthrene removal efficiency, a) Pareto chart and b) the main effect plot.**

The importance and effectiveness of each macro or micro nutrient and its positive or negative effect on removal efficiency is shown in Figure 2a (Pareto chart) and b (the main effect). For factors affecting the process positively, the nitrogen source showed the most importance followed by phosphorus sources; trace elements solution, Zn, FeSO_4_, Mn and mg. Factors affecting the phenanthrene removal negatively included FeCl_2_, NaCl and CuSO_4_.

### Analysis of ANOVA

The ANOVA table partitions the variability in removal into separate pieces for each of the effects (Table 4). It then tests the statistical significance of each effect by comparing the mean square against an estimate of the experimental error. In this case, 11 effects have P-values less than 0.05, indicating that they are significantly different from zero at the 95.0% confidence level.

**Table 4:** **Analysis of variance for phenanthrene removal**

**Source**	**Sum of squares**	**Df**	**Mean square**	**F-ratio**	**P-value**
A:Factor_A	2836.69	1	2836.69	5663.94	0.0000
B:Factor_B	2120.02	1	2120.02	4232.99	0.0000
C:Factor_C	13608.1	1	13608.1	27170.85	0.0000
D:Factor_D	46.0208	1	46.0208	91.89	0.0000
E:Factor_E	756.841	1	756.841	1511.16	0.0000
F:Factor_F	196.021	1	196.021	391.39	0.0000
G:Factor_G	1230.19	1	1230.19	2456.28	0.0000
H:Factor_H	159.141	1	159.141	317.75	0.0000
I:Factor_I	50.8408	1	50.8408	101.51	0.0000
J:Factor_J	521.401	1	521.401	1041.07	0.0000
K:Factor_K	1516.5	1	1516.5	3027.96	0.0000
Total error	18.03	36	0.500833		
Total (corr.)	23059.8	47			

R-squared = 99.9218 percent; R-squared (adjusted for df) = 99.8979 percent; Standard Error of Est. = 0.707696.

Mean absolute error = 0.50625; Durbin-Watson statistic = 3.14504 (P = 0.9982).

Lag 1 residual autocorrelation = −0.573073.

The R-Squared statistic indicates that the model as fitted explains 99.9218% of the variability in removal. The adjusted R-squared statistic, which is more suitable for comparing models with different numbers of independent variables, is 99.8979%. The standard error of the estimate shows that the standard deviation of the residuals is 0.707696.

### The phenathrene removal in optimized conditions for naturally contaminated soil

**Figure 3:**
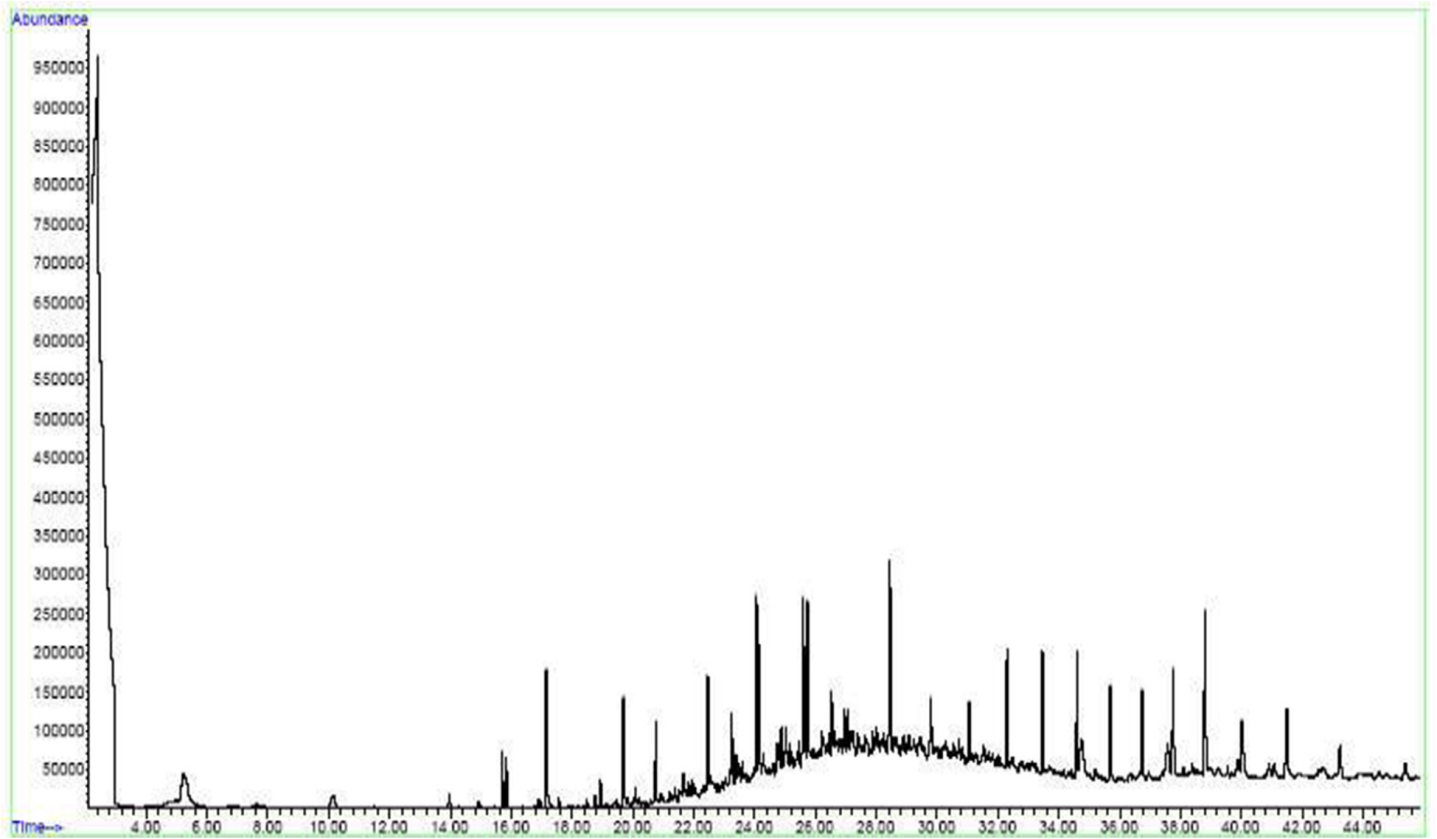
**The GC-MS analysis of naturally contaminated soil.**

A soil sample naturally contaminated to different hydrocarbons was used to investigate to optimized process efficiency for PAHs removal. The GC-MS analysis on the soil sample is presented in Figure 3. According to analysis the picks of GC-MS, different hydrocarbons and four PAHs (to phenenthrene, pyrene, anthracene, flourene) were detected in the soil. The same inoculums like section 2–2 and optimized culture conditions were applied on the samples during 8 weeks. Results are presented in Table 5. The initial phenenthrene, pyrene, anthracene, flourene concentrations were 72, 61, 92 and 46 mg/kg, respectively. After 8 weeks, the phenenthrene, pyrene, anthracene, flourene concentrations were decreased to 31, 29, 26 and 27 mg/kg, respectively. The most removal efficiency of 71.7% was observed for antheracene, followed by 56.9% for phenanthrene, 52.4% for pyrene and 41.3 for flourene. The lower removal efficiency can be referred to the presence of other hydrocarbons and interfering factors of natural soil.

**Table 5:** **The PAHs removal efficiency in naturally contaminated soil**

**PAH**	**Initial concentration (mg/kg)**	**Final concentration (mg/kg)**	**Removal (%) in run 1**	**Removal (%) in run 3**	**Removal (%) in run 4**
Phenanthrene	72	31	56.9 ± 2.3	41.2 ± 1.4	30.5 ± 1.6
Pyrene	61	29	52.4 ± 2.6	37.5 ± 3.6	33.6 ± 2.8
Anthracene	92	26	71.7 ± 1.9	68.7 ± 2.5	50.3 ± 3.4
Flourene	46	27	41.3 ± 3.3	25.2 ± 4.2	22.8 ± 2.7

## Discussion

Bioremediation is often limited by environmental, physical and chemical factors. One of the most important problems in the bioremediation of PAHs is nutrients limitation in the soil and sediment. Biostimulation via addition of inorganic nutrients has been used as a strategy to enhance the biodegradation rate of PAH contaminated soils. Since the optimum values of macro and micro nutrient is highly dependent on the type of contaminant, microbial consortium and soil conditions, there isn’t a unique and clear explanation in the literature about the type and concentrations of nutrients for bioremediation of soils contaminated by hydrocarbons. Therefore, the most effective macro and micro nutrients and their concentrations for each specific application should be determined separately to enhance the removal efficiency of hydrocarbons present in the soil. In the present study, the addition of three macro nutrients, eight micronutrients and four trace elements in two high and low levels (according to literature) was investigated to optimize the combination of these three categories of nutrients. Comparison with similar blanks showed that nutrient biostimulation through nutrients enhanced the phenanthrene removal from soil slurry and this was proved by the previous studies [25-27]. The highest of phenanthrene removal efficiency was observed in run 1 with the presence of high level of KH_2_PO_4_ and NH_4_NO_3_ in mineral salt medium. The second run with the best removal efficiency was run 4 with high level of the same macronutrient as run 1. In these two runs the micro nutrients and trace elements conditions were not in the same form showing the importance of factors C and A as the macronutrients in phenanthrene biodegradation. A relation between mineralization rates of phenanthrene and the initial concentrations of nitrogen and phosphorus as macronutrient has been reported before [28]. Da Silva et al. (2009) reported the improvement in bioremediation of coastal sand contaminated to crude oil by using commercial mineral NPK fertilizer [29]. Borresen and Rike (2007) showed that the concentration of phenanthrene in the soil amended with NP and biosolid was 1.7 and 2.9 times lower, respectively [30].

The relevant effects of eleven factors were sorted from the highest to the lowest in the Pareto chart presented in Figure 2a. The Pareto chart showed that A, B, C, D, G, H, J and K had positively affected phenanthrene degradation, whereas E, F and I had negative effects. All of these factors are in the right side of the t-value line in this chart showing the significant effect of them.

The positive effect of factors shows that the higher concentration of them has more efficiency in biodegradation. Among the eleven, eight factors had the positive effect on phenanthrene removal which confirms the biostimulation of phenanthrene biodegradation by nutrient addition, but Braddock et al. (1997) in their research for bioremediation of hydrocarbon-contaminated arctic soils showed the most biostimulation in the less concentration of nutrients [31]. It may be related to growth inhibition in N and P rich soils [32]. An initial inhibition of bacterial growth by magnitude of 2 Log was seen in the population of consortium in the runs with high concentration of N and P, but it didn’t take too long. Besides in the other of our research with pure culture of bacteria, this reduction was about 3 Log. Lower inhibition of bacterial growth in the mix culture showed different responses for the diverse bacterial populations against to the environmental condition [32] and a need for optimum composition of macro, micro and trace nutrients. If the suitable ratio of nutrients does not supply, the key degrader microorganisms may become ineffective which leading to less progress in biodegradation [33]. After a short time the population of bacterial consortium increased and the maximum density was observed in the runs with higher removal efficiency which may be resulted from a more need of degrader bacteria to the nutrients composition provided in these runs.

Also in the Pareto chart, C was the most effective factor followed by showing the high effect of nitrogen and phosphorus respectively. Olaniran et al. (2006) and Margesin and Schinner (1999) reported the more biotransformation was seen in using nitrogen and phosphorus as fertilizer [34,35]. The main effect plot (Figure 2b) confirms the most effect of macronutrients of C and A too. The slope of NH_4_NO_3_ effect shows that the response of phenanthrene removal was sensitive to this factor regarding to dominant effect of nitrogen in biodegradation. Nitrogen in the form of NH_4_^+^ or NO_3_^−^ is readily assimilated in bacterial metabolism [17]. Ferandez-Luqueno et al. (2009) showed that degradation of poly acryl amide caused to release of nitrogen which leading to increment of the concentration of it and promotion in PAH removal [36]. But Liebeg and Cutright (1999) in their investigation of the effect of macro/micro nutrient, reported that phosphorus was the dominant nutrient in PAH bioremediation [17]. The composition of the best run in their report was consisted of 3% nitrogen, 11% phosphorus and 75% sulfur. In our experiment the runs with 3-12% phosphorus on a dry weight basis had the higher efficiency but phosphorus was not the dominant nutrient. The amount of nitrogen in the runs with more biodegradation of phenanthrene (runs: 1, 3, 4, 6 and 7) was in the range of 67-87%. The analysis of variance confirms that factor C (as NH_4_NO_3_) with the effect of 59% was the most effective nutrient in phenanthrene biodegradation. According to Cookson (1995) the concentration of 150 mg of nitrogen and 30 mg phosphorus has been required for degradation of one gram of a theoretical hydrocarbon into cellular material [37]. Betancur – Galvis et al. (2006) used nitrogen in the concentration of tenfold of phosphorus in biostimulation of PAH contaminated saline–alkaline soils [14]. Atagana et al. (2003) showed more removal of creosote in biostimulation of contaminated soil with lower amount of nitrogen but the more microbial growth was in the higher amount of it [38].

The negative effect of iron and copper (factors E and I) may be related to the lack of requirement of them by the dominant biodegrader bacteria in this experiment [17]. The need for micronutrient or trace element is very different in diverse microorganisms. In our study nutrient solution with composition of: 75% N, 10% P and 14% K was the best mineral salt medium for phenanthrene biodegradation.

Analysis of variance showed that after the factors A and B with the effect of 12.3 and 9.2%, factor C is the most effective factor among the cosidred variables. The total effect of macro, micro and trace nutrients were 80.5, 12.9 and 6.6%, respectively.

The slope of the parameters in the main effect plot showed that all of them had significant effect in the process and the analysis of ANOVAs indicating that they are significantly different from zero at the 95.0% confidence level too. The R-Squared statistic indicates that the model as fitted, explains 99.9218% of the variability in removal. The optimal settings of the experimental factors have been determined and are displayed in the summary in Table 6. Plackett-Burman design has great potential for screening of several variables by assessing the relative importance of these parameters.

**Table 6:** **Factor settings at optimum conditions determines by Plackett-Burman design**

**Factor**		**Setting**	
Factor_A	KH_2_PO_4_	0.996965	2.99 ( g/L)
Factor_B	K_2_HPO_4_	−0.850538	0.6868 ( g/L)
Factor_C	NH_4_NO_3_	0.997797	6.98 ( g/L)
Factor_D	MgSO_4_	−0.995396	0.1007 ( g/L)
Factor_E	FeCL_3_	−0.996413	0.0103 ( g/L)
Factor_F	NaCl	−0.99606	0.011556 ( g/L)
Factor_G	ZnSO_4_.H_2_O	0.997174	0.0499576 (mg/L)
Factor_H	MnSO_4_.H_2_O	0.984212	3.96858 (mg/L)
Factor_I	CuSO_4_.5H_2_O	0.879905	0.37718 (mg/L)
Factor_J	FeSO_4_.7H_2_O	−0.958447	0. 0305687 (mg/L)
Factor_K	H_3_BO_3_	0.999567	12.998 (mg/L)
	Na_2_MoO_4_		0.999 × 10 ^−2^ (mg/L)
	CoCl_2_		0.1 (mg/L)
	NiCl_2_		0.2 (mg/L)

## Conclusion

Biostimulation of PAHs contaminated soils through nutrient addition enhance the biodegradation rate in the process. Our result on statistical screening of media components by Plackett–Burman design proved the advantages of selecting significant media components while phenantrene biodegradation with a bacterial consortium was investigated. The suitable conditions for phenanthrene removal were: as g/L 6.98 NH_4_NO_3_, 2.99 KH_2_PO_4_, 0.6868 K_2_HPO_4_, 0.1007 MgSO_4_,0.0103 FeCL_3_, 0.011556 NaCl, and as mg/ L 12.998 H_3_BO_3_, 3.96858 MnSO_4_.H_2_O,0.37718 CuSO_4_.5H_2_O, 0.2 NiCl_2_, 0.1 CoCl_2_, 0.0499576 ZnSO_4_.H_2_O, 0. 0305687 FeSO_4_.7H_2_O and 0.999 × 10 ^−2^ Na_2_MoO_4_. Plackett–Burman design has good potential for preliminary optimization and more accurate quantitative analysis of the effect of great number of variables for phenanthrene biodegradation.

## References

[1] Gan S, Lau E, Ng H (2009). Remediation of soil contaminated with PAHs. J Hazard Mater.

[2] Jorfi S, Rezaee A, Moheb-ali G, Jaafarzadeh HN (2013). Pyrene removal from contaminated soils by modified Fenton oxidation using iron nano particles. J Environ Health Sci Eng.

[3] Que C, Zhou H, Wong Y, Tam N (2005). Isolation of PAH-degrading bacteria from mangrove sediments and their biodegradation potential. Mar Pollut Bull.

[4] Ni H, Zhou W, Zhu L (2014). Enhancing plant-microbe associated bioremediation of phenanthrene and pyrene contaminated soil by SDBS-Tween 80 mixed surfactants. J Environ Sci.

[5] Huang XD, El-Alawi Y, Penrose DM, Glick BR, Greenberg BM (2004). A multi-process phytoremediation system for removal of polycyclic aromatic hydrocarbons from contaminated soils. Environ Pollut.

[6] Ferrarese E, Andreottola G, Oprea L (2008). Remediation of PAH-contaminated sediments by chemical oxidation. J Hazard Mater.

[7] Zhang L, Li P, Gong Z, Li X (2008). Photocatalytic degradation of polycyclic aromatic hydrocarbons on soil surfaces using TiO_2_ under UV light. Hazard Mater.

[8] Yeung A, Gu Y (2011). A review on techniques to enhance electrochemical remediation of contaminated soils. J Hazard Mater.

[9] Pope C, Peters W, Howard J (2000). Thermodynamic driving forces for PAH isomerization and growth during thermal treatment of polluted soils. J Hazard Mater.

[10] Berezina N, Yada B, Lefebvre R (2015). From organic pollutants to bioplastics: insights into the bioremediation of aromatic compounds by Cupriavidus necator. New Biotechnol.

[11] Yu X, Wu S, Wu F, Wong M (2011). Enhanced dissipation of PAHs from soil using mycorrhizal ryegrass and PAH-degrading bacteria. J Hazard Mater.

[12] Jorfi S, Rezaee A, Moheb-ali G, Jaafarzadeh N (2013). Application of biosurfactants produced by pseudomonas aeruginosa SP4 for bioremediation of soils contaminated by pyrene. Soil Sediment Contam.

[13] Nikolopoulou M, Kalogerakis N (2008). Enhanced bioremediation of crude oil utilizing lipophilic fertilizers combinedwith biosurfactants and molasses. Mar Pollut Bull.

[14] Betancur-Galvis LA, Alvarez-Bernal D, Ramos-Valdivia AC, Dendooven L (2006). Bioremediation of polycyclic aromatic hydrocarbon-contaminated saline–alkaline soils of the former Lake Texcoco. Chemosphere.

[15] Nam K, Kim J (2002). Role of loosely bound humic substances and humin in the bioavailability of phenanthrene aged in soil. Environ Pollut.

[16] Bailey JE, Ollis DF (1986). Biochemical Eng. fundamentals.

[17] Liebeg E, Cutright T (1999). The investigation of enhanced bioremediation through the addition of macro and micro nutrients in a PAH contaminated soil. Int Biodeterior Biodegrad.

[18] Chauhan K, Trivedi U, Patel K (2007). Statistical screening of medium components by Plackett–Burman design for lactic acid production by Lactobacillus sp. KCP01 using date juice. Bioresour Technol.

[19] Zhou J, Yu X, Ding C, Wang Z, Zhou Q, Pao H, Cai W (2011). Optimization of phenol degradation by Candida tropicalisZ-04 using Plackett-Burman design and response surface methodology. J Environ Sci.

[20] Nasseri S, RezaeiKalantary R, Nourieh N, Naddafi K, Mahvi A, Baradaran N (2010). Influence of bioaugmentation in biodegradation of PAHs-contaminated soil in bio-slurry phase reactor. Iran J Environ Health Sci Eng.

[21] RezaeiKalantary R, Badkoubi A, Mohseni-Bandpi A, Esrafili A, Jorfi S, Dehghanifard E (2013). Modification of PAHs biodegradation with humic compounds. Soil Sediment Int J.

[22] Khosravi-Darani K, Zoghi A (2008). Comparison of pretreatment strategies of sugarcane baggase: experimental design for citric acid production. Bioresour Technol.

[23] Kim I, Park J, Kim K (2001). Enhanced biodegradation of polycyclic aromatic hydrocarbons using nonionic surfactants in soil slurry. Appl Geochem.

[24] USEPA: **Polycyclic Aromatic Hydrocarbons (PAHs).** 2008 http://www.epa.gov/osw/hazard/wastemin/priority.htm.

[25] Mills MA, Bonner JS, Page CA, Autenrieth RL (2004). Evaluation of bioremediation strategies of a controlled oil release in a wetland. Mar Pollut Bull.

[26] Coulon F, Pelletir E, Gourhant L, Delille D (2005). Effects of nutrient and temperature on degradation of petroleum hydrocarbons in contaminated sub-Antarctic soil. Chemosphere.

[27] Yu K, Wong A, Yau K, Wong Y, Tam N (2005). Natural attenuation, biostimulation and bioaugmentation on biodegradation of polycyclic aromatic hydrocarbons (PAHs) in mangrove sediments. Mar Pollut Bull.

[28] Teng Y, Luo Y, Ping L, Zou D, Li Z, Christie P (2010). Effects of soil amendment with different carbon sources and other factors on the bioremediation of an aged PAH-contaminated soil. Biodegradation.

[29] Da Silva AC, de Oliveira S, Bernardes DS, de França FP (2009). Bioremediation of marine sediments impacted by petroleum. Appl Biochem Biotechnol.

[30] Borresen MH, Rike AG (2007). Effects of nutrient content, moisture content and salinity on mineralization of hexadecane in an Arctic soil. Cold Reg Sci Technol.

[31] Braddock J, Ruth M, Catteral P, Walworth J, Mc Carthy K (1997). Enhancement and inhibition of microbial activity in hydrocarbon-contaminated arctic soils: implications for nutrient-amended bioremediation. Environ Sci Technol.

[32] Ruberto L, Vazquez S, Mac Cromack W (2003). Effectiveness of the natural bacterial flora, biostimulation and bioaugmentation on the bioremediation of a hydrocarbon contaminated Antarctic soil. Int Biodeterior Biodegrad.

[33] Smith V, Graham D, Cleland D (1998). Application of resource-ratio theory to hydrocarbon biodegradation. Environ Sci Technol.

[34] Olaniran A, Pillay D, Pillay B (2006). Biostimulation and bioaugmentation enhances aerobic biodegradation of dichloroethenes. Chemosphere.

[35] Margesin R, Schinner F (1999). Biological decontamination of oil spills in cold environments. J Chem Technol Biotechnol.

[36] Fernandez-Luqueno F, Thalasso F, Luna-Guido M, Ceballos-Ramirez J, Ordoñez-Ruiz I, Dendooven L (2009). Flocculant in wastewater affects dynamics of inorganic N and accelerates removal of phenanthrene and anthracene in soil. J Environ Manage.

[37] Cookson J (1995). Bioremediation engineering: design and application.

[38] Atagana H, Haynes R, Wallis F (2003). Optimization of soil physical and chemical conditions for the bioremediation of creosote-contaminated soil. Biodegradation.

